# Communication Partners' Journey through Their Partner's Hearing Impairment

**DOI:** 10.1155/2013/707910

**Published:** 2013-02-27

**Authors:** Vinaya K. C. Manchaiah, Dafydd Stephens, Thomas Lunner

**Affiliations:** ^1^Centre for Long Term and Chronic Conditions, College of Human and Health Sciences, Swansea University, Swansea SA2 8PP, UK; ^2^Linnaeus Centre HEAD, Swedish Institute for Disability Research, Department of Behavioural Sciences and Learning, Linköping University, 58183 Linköping, Sweden; ^3^Department of Psychological Medicine & Neurology, School of Medicine, Cardiff University, Cardiff CF14 4XN, UK; ^4^Eriksholm Research Centre, Oticon A/S, 20 Rørtangvej, 3070 Snekkersten, Denmark

## Abstract

The objective of this study was to further develop the Ida Institute model on communication partners' (CPs) journey through experiences of person with hearing impairment (PHI), based on the perspectives of CPs. Nine CPs of hearing aid users participated in this study, recruited through the Swansea hearing impaired support group. Semi-structured interviews were conducted, the data were analysed using qualitative thematic analysis and presented with the use of process mapping approach. Seven main phases were identified in the CP journey which includes: (1) contemplation, (2) awareness, (3) persuasion, (4) validation, (5) rehabilitation, (6) adaptation, and (7) resolution. The Ida Institute model (based on professionals' perspective) was compared with the new template developed (based on CPs' perspectives). The results suggest some commonalities and differences between the views of professionals and CPs. A new phase, adaptation, was identified from CPs reported experiences, which was not identified by professionals in the Ida Institute model. The CP's journey model could be a useful tool during audiological enablement/rehabilitation sessions to promote discussion between the PHI and the CP. In addition, it can be used in the training of hearing healthcare professionals.

## 1. Introduction

Communication partners (CPs) are those with whom the person with hearing impairment (PHI) communicates on a regular basis. The term communication partner has been used to refer to the significant others which may include their spouse, siblings, children, friends, relatives, colleagues, and carers. 

Hearing impairment is a communication problem which affects everyone in the communication situation, not only the PHI [1]. It can result in various physical, mental, and psychosocial effects on PHI and their CPs. According to the World Health Organisation-International Classification of Functioning, Disability, and Health (WHO-ICF), spouses of PHI, although they do not have a health condition themselves, may experience activity limitations and participation restrictions due to their spouses' health condition which is referred to as a “third-party disability” [2]. Studies have shown that CPs may undergo various experiences through their partners' hearing loss, and this may often influence the help-seeking behaviour of the PHI [3–6]. Our recent review identified various impacts that CPs can have due to their partners' hearing loss, and suggesting the need to involve CPs in the audiological enablement/rehabilitation which will result in mutual advantages for both the PHI and their CPs [7]. Moreover, exploring the journey of CPs through the PHI's hearing loss was identified as one of the key research questions. 

Ida Institute at Denmark is a non-profit organisation with a mission to foster better understanding of human dynamics of hearing loss. The institute conducts various activities to create and share innovative, actionable knowledge to help hearing care professionals address the psychological and social challenges of hearing loss and implement patient-centered care practices. The institute with collaborative effort from hearing healthcare professionals around the world developed the possible CP journey model [8]. However, this model was based only on professionals perspectives and includes six main phases: (1) what is going on?, (2) awareness, (3) persuasion, (4) validation, (5) rehabilitation, and (6) maintenance. Further details of this model are presented in Section 3. It was suggested that this model/template recognises the emotional reactions and practical activities the CPs experience during the onset of their partner's hearing loss, successful management and learning to live with the condition. However, studies from medical anthropologists and also from our previous studies on patient journey of PHI have shown differences in professionals' and patients' perspectives [9–11]. This may indicate that the professionals' and the CPs' perception of the CP journey could be different. 

In a recent international study that focused on exploring the perspectives of the PHI, it was highlighted that PHI use their life experiences rather than clinical encounters to describe the hearing help-seeking and hearing rehabilitation process [12]. In addition, it was suggested that the patients did not report their experiences on clinical encounters towards hearing help-seeking and rehabilitation as a connected process. This may be because they may have not made an attempt to think about the help-seeking and hearing rehabilitation in the temporal order, and to some extent, they may have even forgotten some of the experiences. Whilst there are studies focusing on the impact of the person's hearing impairment on CPs [7, 13, 14], we were unable to identify any studies that focused on the perspectives and experiences of CPs through their partner's hearing help-seeking and rehabilitation process. In addition, we believe that such an effort to map the process over time and understand the journey of CPs may give some insights into how CPs are affected by PHIs' hearing loss, how they might cope with these effects, and how they may influence the journey and the help-seeking behaviour of the PHI. Such journey models of PHIs and CPs could be helpful during the audiological enablement/rehabilitation process to prompt the discussion between PHIs and CPs and potentially in developing the relationship-centered care (RCC). 

**Figure 1:**
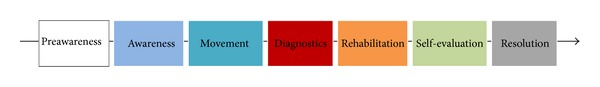
Patient's journey model of adults with gradual-onset acquired hearing impairment [10].

In our previous studies, we have explored the journey of PHI [10, 11].  Figure 1 shows the typical patient's journey model of adults with gradual-onset acquired hearing impairment which has seven main phases: (1) preawareness, (2) awareness, (3) movement, (4) diagnostics, (5) rehabilitation, (6) self-evaluation, and (7) resolution. These phases correspond quite well with stages of the transtheoretical model of change which demonstrates individual's readiness to act on a new health behaviour [15, 16]. 

The aim of the current study was to further develop the Ida Institute model on the CP journey through their partner's hearing impairment, based on the perspectives of CPs and to examine the relationship between the perspectives of hearing healthcare professionals and of CPs.

## 2. Method

### 2.1. Participants

Ethical clearance was obtained from Departmental Research Ethics Committee, College of Human and Health Sciences, Swansea University. A purposeful sampling strategy [17] (usually used in theory-driven or theory-developing qualitative research) was used in order to recruit CPs of the hearing aid users through the Swansea Hard of Hearing Support Group. Most members of this group are healthcare users of National Health Services, experienced hearing aid users (i.e., over 2 years), and they meet once a month for few hours in a designated place. The participants included 9 CPs which involved spouses, children, friends, colleague and a carer. Whilst the participants include a wide range of people, all were reported to spend a significant amount of time communicating with the PHI (i.e., during most days of the week). However, CPs typically do not attend the support group meetings.  Table 1 presents the demographic details of participants. There were 7 females and 2 males with a mean age of 44.4 years (ranging from 19 to 74 years). The duration of contact of the CP with the PHI varied from 1 year to 58 years with an average of 25.6 years and the duration of PHI's hearing loss varied from 6 years to >30 years. Only 3 of the 9 CPs in the study had accompanied the PHI to an audiological appointment on at least one occasion. All PHI were reported to have bilateral hearing loss with mild to severe degree. However, we were unable to obtain the exact degree of hearing loss in each case. 

### 2.2. Data Collection

All the participants were supplied with an information sheet usually a week before the interview and scheduled an appointment. In addition, on the day of interview, they were given a short introduction to the study, an opportunity was given to ask questions, they were informed about confidentiality, and a written consent was obtained. The data were collected through semistructured interviews. A questionnaire was developed based on the literature review and from our previous experience of studies on the journey of PHI, which was used as a guide during the interview (see the appendix for details). Initially, CPs were asked to narrate their journey (tell their story) through their partner's/friend's/father's hearing loss. This was followed by some general questions (i.e., all the questions in the questionnaire were asked to each participant) to explore the CPs' experiences broadly. In addition, more directed questions based on their reports during the interview were asked to obtain an in-depth understanding of their experiences. Interviews typically lasted for about 60–90 minutes. The interviews were recorded using portable digital recorders to recheck the notes taken by the researcher. It was noticed that many CPs had prepared notes about their experiences before the interview, even though it was not requested in the information sheet. 

### 2.3. Data Analysis

The data collection and the data analysis were conducted by the first author. Thematic analysis which involves identifying, analyzing, and reporting patterns within the data was employed to analyse the data [18]. The main task in thematic analysis is to identify a limited number of themes which adequately reflect the data. A hybrid of inductive and deductive approaches was used for the coding and the development of themes [19]. The Ida Institute CP's journey model was used as a theoretical base [8]. Such an approach allowed the researcher to focus on important aspects of the data based on theory and also to look for new themes which emerged from the data. 

The main steps in data analysis included: familiarization with the data by repeated reading of notes and by listening to the voice recordings repeatedly, generating the initial codes (i.e., the participants' reports were shortened to simple and meaningful units), categorising the data and searching for subthemes and themes, ongoing review, defining and naming of themes and subthemes (the Ida model acted as an inspiration for naming the themes), and identifying some extracts which could be used in reporting the data. The subthemes were categorised into most (i.e., approximately two thirds), many (i.e., approximately half), several, and/or few (i.e., less than half) based on how frequently they were reported by the participants. Moreover, the rule of most of participants reporting was considered for a theme (i.e., phase) and many participants reporting for a subtheme (i.e., stage). The working model with seven main phases was developed with the interview data of seven participants. Two new participants were interviewed to check for data saturation, and the data collection was stopped as there were no new themes (i.e., phases) being identified (i.e., data saturation—no significant new data emerging from the data in relation to research question) [20, 21].

A total of 58 unique subthemes that were related to the study were identified through 9 interviews. However, only 31 of them that were reported by most and many participants were considered for the development of CP's journey model. An ongoing matching of subthemes was done with Ida Institute model to see if the same code names can be assigned. However, where new subthemes (i.e., stages) were identified, new names were assigned to reflect the meaning and essence of the reported experiences. The subthemes were grouped together to identify themes, and the themes were further confirmed by repeatedly listening to participants' interviews to check if the identified themes capture the reported experiences. The process mapping (i.e., a way of representing a sequence of actions involved in a process) was used to define these themes in appropriate phases to represent the CP's journey model [22]. Process maps can be an effective way to demonstrate either individual or organisational process about virtually any aspect. The visual approach used in presenting the information makes it easier for readers to understand the process and may also help in identifying any constraints and/or bottle necks. Whilst the use of process mapping in healthcare seems to be relatively new, it has increased mainly in clinical audits to identify how we manage the patient's journey, using patient's perspectives to identify issues and suggested improvements to healthcare [23, 24]. Such an approach to presenting qualitative data about the patient's journey has also been used in our previous studies and also by others [10, 11, 25]. 

## 3. Results

**Figure 2:**
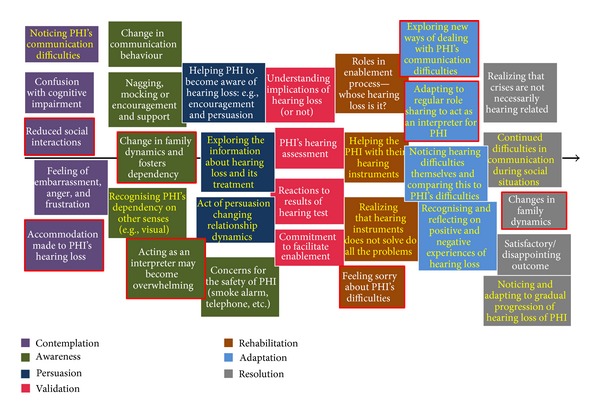
Communication partner's perspectives of their journey through their partners' hearing impairment (stages identified only by CPs are highlighted in yellow text, and stages which are reported in multiple phases are highlighted with red outline).

Seven main phases and various stages were identified.  Figure 2 shows the CPs' perspective on their journey through their partners' hearing loss. In this section, we present the phases and stages of the CP's journey in a logical order. Whilst there was some temporal order to participants' narratives, not all the CPs reported them in this order. For example, many participants went back and forth while talking about a particular theme. However, those who consistently maintained the temporal order are the ones who usually had prepared notes for this interview after reading the participant's information sheet. This may suggest that there is generally a temporal order to participants' reported stories; however, their ability to remember the fine details and articulate the experiences may have influenced this. Based on data from this study and also our previous studies on patient's journey, we suggest that there is a journey through this process. For this reason, we decided to present them in a linear fashion using process mapping, even though not all reported them in such a systematic manner. In addition, some stages were reported in more than one phase. For example, role sharing and relationship dynamics that were reported both in initial and later phases of the journey. CPs talked more about contemplation, awareness, adaptation, and resolution phases compared to other phases (i.e., persuasion, validation, and rehabilitation). Moreover, the reported experience of one of the CPs (participant 9) was quite unique compared to the others. In this case, the CP had grown up through her father's hearing loss rather than starting to notice the hearing loss when the PHI initially started developing it. In that case, whilst she did not report a contemplation stage, she reported how she started becoming aware of her father's hearing loss as she got older and also the reported experiences in all the other phases. 

### 3.1. Contemplation (or What Is Going On?)

In this phase, CPs may start noticing the PHI's communication difficulties and reduced social interactions. This may sometimes result in feeling embarrassed, angry, and frustrated. Initially, the CPs might attribute some of the problems noticed to possible cognitive impairments, attentions and concentration. Moreover, the CPs may also start making some accommodation, to the PHI's hearing loss. 

The following statement made by the CP of a PHI shows how, in the initial phase of the PHI's hearing loss, the CP may think that the communication difficulties noticed were due to attention and concentration rather than to poor hearing. This highlights the fact that the identification of hearing loss is not straightforward.

*Initially I thought a lot of it was due to his attention…! I could say something to him and if it was not of his interest, I could see that he has not heard it, or he will repeat what I said five minutes later, and I would say…..I just told you that..! …. Even though I had experience with deafness due to others in the family, I could not realise he had problem straightaway. *

### 3.2. Awareness

In this phase, CPs become aware that the PHI has genuine difficulties with their hearing. This may be by noticing clear changes in the PHI's communication behaviour, PHI's dependency on other senses, noticing that PHI was not hearing the smoke alarm, telephone, and so forth, and more importantly, by noticing changes in the family dynamics. They may start nagging the PHI (or indirectly persuading the PHI to seek help) or provide support and encouragement, and they may start acting as an interpreter for the PHI. However, this new role of acting as an interpreter may become overwhelming. 

This description below shows how a CP confirmed their speculation about the PHI's hearing loss (elements of contemplation and awareness phases). It also highlights how this awareness may change the family dynamics.

*I can remember a few things which can put the picture together…The first thing I noticed was that he was shouting on the telephone. I could be in there with the doors shut and could hear him. I say to him, do you realise that you were shouting on the telephone?, I do not think I was.. You cannot say anything to that one…… and the other thing is shouting at public places, for example, shouting in the restaurant. *

*….after it was confirmed to me with these observations, I told my children what I noticed and they agreed, especially the elder daughter, and she started making some adjustments… *

The following statement made by a CP highlights the change in their communication roles, a change in family dynamics and the dependency of the PHI on the CP for everyday activities in relation to communication.

*After I started noticing his difficulties…I almost started acting like his secretary…it could be very tiring sometimes…especially later in the day…!!*

### 3.3. Persuasion

After CPs become aware of the PHIs hearing loss, they often start making attempts to make the PHI aware of their communication problems. In addition, they may also start searching for information related to hearing loss and start persuading them to seek help. In the initial stages, this could be indirect. However, there could be some triggering factors for the CPs which make them start directly persuading the PHI to seek help. 

The following quote confirms that the CPs could act as drivers (or facilitators) to the PHI seeking help. The CP's expression in this makes it clear that this task is not always straightforward. They may start with indirect persuasion and move to more direct persuasion as time progresses.

*I have to be very diplomatic you know. ….. [Chuckle]…. I got a bit of adverse reaction on one occasion. He said to me speak up you are mumbling, and I said to him you are not hearing me properly and asked him to get his hearing checked. ….. [Chuckle]…. He said to me ‘you get your hearing checked', you don't hear something that I say to you… Once he said that I have to back off for a while obviously….work with it for a while and then change my approach…*

*…….I think I nagged him to such an extent that he went to get a hearing test… he was not hearing the telephone, once he did not hear the alarm and ……once it got to that stage I have to tell him..!!*

### 3.4. Validation

This phase was not widely discussed by the CPs. However, in this phase, CPs mainly confirm whether or not the PHI had hearing loss. The results of hearing assessment of the PHIs may or may not surprise the CPs. Even though most CPs were not very keen about the hearing assessment, some accompanied the PHI for hearing assessment and made an attempt to understand the hearing test results and what they may indicate. However, almost all of them made commitments to support the PHIs. 

In this statement, the CP talks about the PHI's reaction to the hearing test and acceptance of hearing loss. However, this also indirectly implies that the CP confirmed that their assessment was correct. There are also elements of later stages being mentioned, for example, the rehabilitation phase (i.e., starting to wear hearing aids).

*After the hearing test, the realisation made him do something about it….after he consulted he started wearing hearing aids, getting them fixed regularly, adjusts them, and it has made a great difference to us.*

### 3.5. Rehabilitation

In this phase, most CPs were relieved that the PHIs were seeking help. However, they started realising that they also have an important role to play in the rehabilitation process, mainly in supporting the PHI (e.g., in using hearing aids). They soon realised that hearing instrument may not solve all the problems, which made them feel sympathy for the PHI's difficulties. 

The following description highlights that soon after the PHI is fitted with hearing aids, they will start realising that they may not solve all the problems. In addition, the coping strategies used and the way in which the CP would support the PHI are evident.

*He wears hearing aids, but I still have to shout and I say things six times….oh….I have to say six times very often..! You hear what I said then?….He will say no.. oh….right….I will start again then. So, it's again my temperament….I don't get cross over him… I would say…oh… for goodness sake…you listening now?….[chuckle]…watch my lips….[chuckle]…*

The following statement made by a CP is an example of what may happen at a dinner table when they have big family dinner. This may suggest that they feel sorry for the PHI as they feel helpless in some occasions.

*I feel a bit guilty sometime….Everyone having a conversation…having a laugh and everything….I feel guilty sometime if he can't join in sometime, and if he is sitting in the corner…and everyone don't realise that.*

### 3.6. Adaptation

This was a new phase identified from CP's reports when compared to the Ida Institute professionals' perspectives of the communication partners' journey [8]. This phase was noticed soon after the hearing assessment and rehabilitation session, when CPs started exploring new ways to communicate with the PHI, adapting to regular role of sharing, and reflecting on positive and negative consequences of the hearing impairment and the audiological management. Many elderly CPs also reported having started noticing hearing problems themselves and started comparing their own problems to those of the PHI. 

The spouse of a PHI made the following statement in relation to how they started exploring new ways of communication after he was confirmed as having hearing loss.

*I do repeat things for him, yes, he said such and such…sometime he will ask to me what did he say, when he misses a bit…the other day I repeated three times and eventually I say, I spell it out…because if they don't get it after three times….then we just spell it out.*

The following quote was made while the CP was talking about how he adapted to dealing with the PHI which demonstrates that the CP is able to identify some positive aspects of hearing loss.

*It's not like he is missing a lot, because we talk after the meeting, what is been said, and it's not like he is missing anything. In fact, there is another person in the office who just can't be bothered, who goes to meeting and ….dreams. Whereas he [name of PHI] is different, he concentrates on what is being said and pays attention. Maybe it's been a benefit to him in that respect, because, he is hard of hearing he got to concentrate on hearing it.*

### 3.7. Resolution

This was a more stable phase which most of the CPs reported during the course of the interview. In this phase, CPs started noticing continued difficulties experienced by the PHI in social situations and started realising that crisis may not necessarily hearing related. They also gradually started noticing the increasing difficulties of the PHI, possibly due to the worsening of their hearing loss. Some CPs had positive temperaments and reported satisfactory outcomes. However, others reported frustrating and disappointing outcomes. Changes in family dynamics (more of relationship dynamics) seemed like a dynamic process which was noticed even in this phase. Moreover, in this phase, most CPs were more stable, and hearing loss had just become a way of life compared to the earlier phase (i.e., adaptation) in which they were exploring new ways of communication to improve the situation. 

The following statement shows that the CP has started using a certain way of communicating rather than exploring new ways of communication.

*We have a way of talking to him now. We have a certain way. We sometime do it with normal hearing people when I talk to them, and they say why are you speaking to me like this? I am so used to being around dad. It does change our lives. *

A daughter of a PHI made this statement indicating how her life had changed, and the crisis was not only hearing related.

*It's our responsibility now…because, we can't expect him to do everyday things now. He will pick up the phone and ring me. Nine out of ten times he does not understand what we say. Few times now, since my mother has died and he lives on his own, if we don't get a response from him on the telephone we have to go there to check if he's alright.*

### 3.8. Other Interesting Observations

A few interesting observations were made while analysing the data. The progression of CPs from one phase to other phase varied in terms of time scale (i.e., a few weeks to a few years). For example, in the case of a carer, there was very limited time between the contemplation and adaptation phase. Moreover, each CP had different expectations of their PHI and most CPs reported that they had taken additional responsibilities after their partner developed hearing impairment (e.g., answering the telephone and interpreting conversations in difficult listening situations). Whilst CPs may need to continually adapt to life situations (as the hearing loss of PHI progressed, changes in the use of technology, etc.), in the initial phases of the journey, CPs reported having explored ways to improve their communication behaviour (i.e., exploring coping strategies). However, as time progressed, they became used to dealing with the PHI rather than finding new ways to improve their communication. Such observations also acted as the key difference between adaptation and resolution phase.

CPs, who reported less psychosocial consequences and who were coping well, appeared to have had a positive temperament (or attitude) towards life and also had some experience of dealing with other chronic conditions. However, this was not measured using any standard scale but only a subjective interpretation of the researcher. More research is needed to understand the relationship between such factors as CP temperament and personality and their influence on the success of audiological rehabilitation of the PHI. 

### 3.9. Comparison to Professionals' Perspectives of the CP's Journey

**Figure 3:**
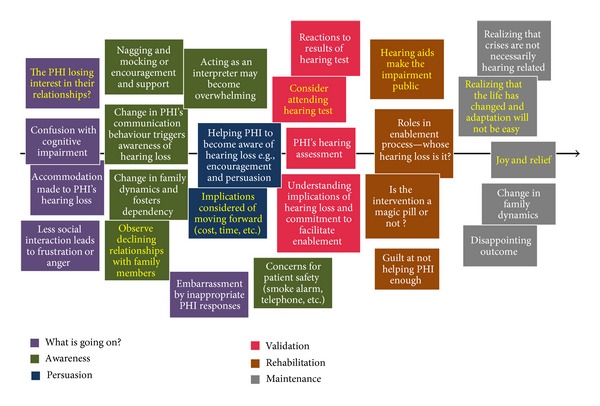
Professionals' perspective of the communication partners' journey (stages identified only by professionals are highlighted in yellow text) [8].

Figure 3 shows the professionals' perspectives of the CPs' journey [8]. This was developed by collaborative efforts of 75 hearing healthcare professionals from around the world who attended the seminars of Enabling Communication Partnerships conducted by the Ida Institute in Denmark during 2009-2010. 

Table 2 shows the differences and similarities in the key phases and/or stages identified by CPs and professionals. This suggests that the unique stages identified only by professionals were relatively few. Moreover, there were some stages which were identified by the professionals but were coded differently when we analysed the experiences reported by CPs. For example, in the contemplation phase, professionals identified that less social interaction leads to frustration or anger. However, CPs reported “reduced social interactions” and “feeling of embarrassment, anger, and frustration.” This is because there were other reasons (e.g., communication breakdown) which also resulted in the feeling of frustration and anger. Moreover, the CPs have highlighted the fact that some stages may occur in more than one phase (e.g., reduced social interactions, changes in family dynamics, and acting as interpreter may become overwhelming). 

## 4. Discussion

**Figure 4:**
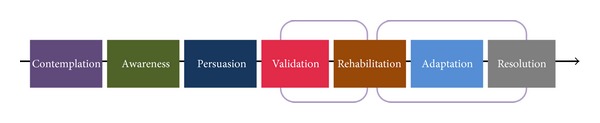
Main phases of communication partners' journey through their partner's hearing impairment (various stages are not drawn to any scale in regard to duration, and time spent in each phase may vary between individuals).

Figure 4 shows the main phases of the CPs' journey through their partners' hearing loss. Seven main phases were identified which include (1) contemplation, (2) awareness, (3) persuasion, (4) validation, (5) rehabilitation, (6) adaptation, and (7) resolution. 

CPs referred more to the initial and later phases in their journey through their partner's hearing loss. Similar results have also been found in studies focusing on experiences of PHI [10, 12]. These findings strengthen the argument that patients and their CPs use their life experiences to relate to the chronic condition rather than the experiences during clinical encounters. This has important clinical implications in that clinicians may have to employ the strategy of talking more about life experiences to find common ground between the PHI and CPs rather than about the disease, clinical tests, and other technical details. The observations, such as CPs having different expectations from the PHI, need more exploration in terms of new ways to improve their communication with the PHI at the beginning of the condition. Moreover, the fine differences between adaptation and resolution phases may highlight the fact that it is important to involve CPs in audiological enablement/rehabilitation at the earliest stage to give them support (i.e., social and emotional) and to teach them communication strategies. Moreover, studies suggest that there is considerable variation in how caregivers adapt to their care-giving demands [26]. For this reason, it is important to better understand CPs' experiences through PHI's hearing loss. 

The study highlights the fact that the professionals failed to identify an important phase adaptation of the CPs' journey. Possible reasons for this may be that the professionals paid less attention to the subjective experiences of the PHI and CP during the rehabilitation process and/or professionals not being able to differentiate between adaptation and resolution phases. Similar results were seen in our previous study on the patient's journey of adults with gradual-onset acquired hearing impairment where professionals did not identify the self-evaluation phase [10]. In addition, some indications of what is described as adaptation and self-evaluation were also seen in studies by Engelund in her thesis which was focused on defining the process of help-seeking in PHI [27]. 

### 4.1. Applications of the Study

The current study has number of clinical and research implications. This is because the model helps to see the bigger picture about experiences of CPs and how they change over time, which is the most important in managing chronic conditions such as hearing impairment. Considering most of the research focuses on specific question (which may only provide fragments of information about CPs experiences), this innovative approach may help organising such information using the proposed model. Whilst the way in which the data presented in this study is quite unique to qualitative research, it appears to be a format which is relatively easy for both professionals and nonprofessionals to understand and remember (i.e., organisation of information chronologically which is the format generally used in storytelling). Furthermore, the study highlights that it is important to understand the perspectives of both professionals and CPs as there are differences and commonalities. 

More specifically, in this study, the data reflected personal stories of CPs through PHI's hearing loss which was used to develop typical journey model of CPs. This may suggest that the narrative (i.e., storytelling/listening) approach appeared to be a simple and useful way to gather data from CPs (similar to our previous study findings on PHIs) [28]. We suggest that this model could be helpful for clinicians to identify which phase (e.g., contemplation, awareness, and persuasion) the CPs might be during clinical encounters by taking in-depth history. This model can be presented to PHIs and CPs to make them think about their journey, and as a starting point in history taking, clinicians could ask CPs to describe their stories. Understanding how the experiences of CPs change over time and what phases CPs are at could be important for clinicians during counselling in order to tailor the information provided to meet the individual CP's needs. Moreover, it is important for hearing healthcare professionals to understand the journey of both the PHI and their CPs in order to facilitate their partnership during audiological enablement/rehabilitation. For this reason, CP's journey model could be used in training hearing healthcare professionals.

Aspects such as the frequency of communication and emotional closeness played an important role in the extent to which the CP was affected by the person's hearing impairment. For example, three of the participants (a carer, a friend, and a colleague) reported very few psychological consequences on them. In addition, three of the CPs (two daughters and a friend) talked about the impact of the PHI on their spouse and expressed the fact that the spouses of PHI were most affected in communication, social, and emotional aspects. This identifies the need for understanding more about the social networks of the PHI and their communication behaviour with CPs. For this purpose, tools such as “communication world” and/or “communication rings” could be helpful [1, 29]. 

In addition, the identification of a new phase (i.e., adaptation phase in the CP's journey, and self-evaluation phase in the PHI's journey from our previous study) [10] is significant in terms of clinical practice which may highlight the need of having review appointments soon after the initial assessment and rehabilitation session. These sessions should be focused on assessing and modifying expectations, providing psychosocial support, and teaching communication tactics to CPs. Moreover, the literature suggests positive outcomes of involving CPs in the audiological rehabilitation sessions [30, 31]. 

### 4.2. Limitations of the Model

In general, the intensity of psychological, emotional, and social consequences reported by each CP varied. For example, to what degree the CPs experienced communication difficulties during social situations. Whilst the CP's journey model represents the main experience of CPs through their partner's hearing loss (i.e., phases and stages) over a period of time, it may not clearly differentiate to what extent individual CPs were affected. This may suggest that this model provides us with an understanding of CPs experiences over time. For this reason, informal questioning, use of open-ended questionnaires, and use of structured questionnaires such as Significant Other Assessment of Communication (SOAC), Hearing Handicap Inventory for Elderly for Spouses (HHIE-SP), and the Significant Other Scale for Hearing Disability (SOS-HEAR) can be useful during clinical encounters to gather information about the effect of the PHI's hearing loss on CPs in different dimensions [32–35]. It is important to note that informal questioning during clinical encounters generally focuses on some elements of CP's and PHI's experiences which may be of clinical interest due to the limited time. Structured questionnaires (e.g., SOAC, HHIE-SP, etc.) focus on the problems experienced at a particular point in time and the intensity to which they are experienced. The use of structured questionnaires before and after treatment and/or management may provide information about the effectiveness of treatment and/or management (i.e., outcome measure). However, the main focus in our approach was to understand how the experiences change over time (i.e., process of change or process evaluation) [36]. For this reason, the combination of such approaches may be necessary in practice, and they may act as complementary to each other. Moreover, this model is one of a variety of ways in which the CP's experiences can be illustrated [8]. Nevertheless, even though this model may not exactly represent each individual CPs' journey, it can be used as a tool to promote discussion with the PHI and CPs to explore their journeys further. 

### 4.3. Advantages and Shortcomings of the Study

The study's methodology has some advantages and drawbacks which may have influenced the results and the development of the model. For example, whilst thematic analysis offers theoretically flexible approach to the analysis of the data, other approaches such as narrative or other biographical approaches may have tapped into different aspects of the data (i.e., being able to retain a sense of continuity and contradiction through any individual account) [18]. However, thematic analysis helped in focusing on specific themes derived from the data and highlighting overlaps in themes in the journey model (i.e., stages appearing in multiple phases). 

Considering the nature of the study, the data collection and analysis were conducted by the first author, and the analyses was discussed with other two authors. This allowed consistency in the method but may have failed to provide multiple perspectives and rechecking the coding. In addition, using notes and voice recordings for data analysis when compared to transcribing the recordings may have some advantages and disadvantages. For example, having the transcription of the interviews may have made the process of coding the data into themes and subthemes easier. However, considering that the data were collected and analysed by the same researcher, we would argue that the researcher was sufficiently familiarised to establish themes and subthemes. Moreover, listening to voice recordings repeatedly rather than looking at the transcriptions allowed the researchers to rethink and reperform parts of the analysis and helped in identifying the differences in intensity of reported psychological, emotional, and social consequences at different points which are not clearly reflected in the proposed model. 

### 4.4. Further Research

The reported experience of CPs may vary, based on differences in cultural aspects, social structures, healthcare structures, educational background, and so on. For this reason, it would be interesting to study the CP's journey from other social and cultural backgrounds. Furthermore, an important question would be to explore how PHI's and their CPs' interactions may influence each other's journey. In the current study, most of the CPs were in the resolution stage and the journey reported was retrospectively based on what they could remember. However, it would be important to investigate the CP's journey longitudinally, by interviewing the CPs at different points of time. More importantly, whilst this exploratory study provides a model of CPs' journey, it should be validated using appropriate quantitative methods on a large sample size. 

## 5. Conclusions

The study highlighted commonalities and differences in perspectives of CPs and professionals. The CP's journey model could be a useful tool during audiological enablement/rehabilitation sessions to promote the discussion between PHI and CPs. In addition, it can be used in training the hearing healthcare professionals. The CP's model was developed from a relatively small sample and may not represent the diverse group of CPs' experiences. Moreover, even though the CP's journey model illustrates CPs' experience through their partner's hearing loss, it may not cover all the complex dimensions. For this reason, the model should be used as a starting point to explore the CP's journey further in clinical situations. 

## Figures and Tables

**Table 1:** Demographic details of communication partners of PHI.

No.	Age	Sex	Relationship with PHI	Duration of contact with PHI	Duration of PHI's hearing loss	Accompanied PHI to audiological session at least on one occasion
1	52	Male	Colleague/friend	15 years	>20 years	No
2	58	Female	Daughter	58 years	>30 years	Yes
3	74	Female	Spouse	48 years	30 years	Yes
4	31	Female	Spouse	4 years	15 years	No
5	75	Female	Spouse	46 years	12 years	No
6	19	Female	Carer	1 year	>10 years	No
7	19	Female	Friend	7 years	>10 years	No
8	53	Male	Spouse	33 years	6 years	No
9	19	Female	Daughter	19 years	>20 years	Yes

**Table 2:** Differences and similarities between phases/stages identified by CPs and professionals.

Phases	Stages identified only by CPs	Stages identified only by professionals	Stages identified by both CPs and professionals
Contemplation (or what is going on?)	Noticing the PHI's communication difficulties	Is the PHI losing interest in their relationship?	Confusion with cognitive impairment; accommodation made to the PHI's hearing loss; less social interaction leads to frustration and anger; embarrassment by inappropriate PHI responses; feeling of embarrassment, anger, and frustration

Awareness	Recognising the PHI's dependency on other senses (e.g., visual)	Observe declining relationships with family members	Nagging and mocking or encouragement and support; changes in the PHI's communication behaviour; changes in family dynamics and fosters dependency; acting as an interpreter may become overwhelming; concerns for the safety of PHI (smoke alarm, telephone, etc.)

Persuasion	Exploring the information about hearing loss and its treatment; act of persuasion changing relationship dynamics	Implications considered of moving forward (cost, time, etc.)	Helping PHI to become aware of hearing loss, for example, encouragement and persuasion

Validation		Consider attending a hearing test	Understanding the implications of hearing loss (or not); commitment to facilitate enablement; PHI's hearing assessment; reactions to results of hearing test

Rehabilitation	Helping the PHI with their hearing instruments; Realising that hearing instruments do not solve all the problems	Hearing aids make the impairment public	Roles in enablement process—whose hearing loss is it? feeling sorry about the PHI's difficulties

Adaptation	Exploring new ways of dealing with the PHI's communication difficulties; adapting to regular role sharing to act as an interpreter for the PHI; noticing hearing difficulties themselves and comparing this to the PHI's difficulties; recognising and reflecting on positive and negative experiences of hearing loss		

Resolution (or maintenance)	Continued difficulties in communication during social situations; noticing and adapting to gradual progression of hearing loss of PHI	Realizing that life has changed and adaptation is not easy; joy and relief	Realizing that crises are not necessarily hearing related; changes in family dynamics; satisfactory/disappointing outcome

## Appendix

### Interview Questionnaire

1. Tell me about yourself and also tell me the story of your journey through your partner's/friend's/father's hearing loss.
2. How did you confirm that your partner/friend/father had hearing loss?
3. What were your immediate reactions to your partner's/friend's/father's hearing loss?
4. What were the reactions of your partner/friend/father towards their hearing loss?
5. What did you and your partner/friend/father think about hearing assessment, management, and rehabilitation sessions? Did you do anything in particular during and/or after these sessions?
6. What life adjustments did you have to make because of your partner's/friend's/father's hearing loss?
7. What effects does the hearing loss of your partner/friend/father have on your quality of life?
8. Have you had any positive experiences due to your partner's/friend's/father's hearing loss?
9. What are your strategies to cope with your partner's/friend's/father's hearing loss?
10. What are the main stages (or important milestones) you went through with your partner's/friend's/father's hearing loss?
